# 1024-channel electrophysiological recordings in macaque V1 and V4 during resting state

**DOI:** 10.1038/s41597-022-01180-1

**Published:** 2022-03-11

**Authors:** Xing Chen, Aitor Morales-Gregorio, Julia Sprenger, Alexander Kleinjohann, Shashwat Sridhar, Sacha J. van Albada, Sonja Grün, Pieter R. Roelfsema

**Affiliations:** 1 Department of Vision & Cognition, Netherlands Institute for Neuroscience, Meibergdreef 47, 1105 BA Amsterdam, Netherlands; 2 Institute of Neuroscience and Medicine (INM-6) and Institute for Advanced Simulation (IAS-6) and JARA Institute Brain Structure-Function Relationships (INM-10), Jülich Research Centre, Jülich, Germany; 3 Institute of Zoology, University of Cologne, Cologne, Germany; 4 Theoretical Systems Neurobiology, RWTH Aachen University, Aachen, Germany; 5 Institut de Neurosciences de La Timone, CNRS & Aix-Marseille University, Marseille, France; 6 Department of Integrative Neurophysiology, VU University, De Boelelaan 1085, 1081 HV Amsterdam, Netherlands; 7 Department of Psychiatry, Academic Medical Center, Postbus 22660, 1100 DD Amsterdam, Netherlands

**Keywords:** Striate cortex, Extrastriate cortex, Neural decoding, Sensory processing

## Abstract

Co-variations in resting state activity are thought to arise from a variety of correlated inputs to neurons, such as bottom-up activity from lower areas, feedback from higher areas, recurrent processing in local circuits, and fluctuations in neuromodulatory systems. Most studies have examined resting state activity throughout the brain using MRI scans, or observed local co-variations in activity by recording from a small number of electrodes. We carried out electrophysiological recordings from over a thousand chronically implanted electrodes in the visual cortex of non-human primates, yielding a resting state dataset with unprecedentedly high channel counts and spatiotemporal resolution. Such signals could be used to observe brain waves across larger regions of cortex, offering a temporally detailed picture of brain activity. In this paper, we provide the dataset, describe the raw and processed data formats and data acquisition methods, and indicate how the data can be used to yield new insights into the ‘background’ activity that influences the processing of visual information in our brain.

## Abstract (summary)

**Table:** 

Measurement(s)	brain activity measurement • eye movement
Technology Type(s)	extracellular electrophysiology recording • multi-electrode array system • infrared camera
Factor Type(s)	visual stimulation
Sample Characteristic - Organism	Macaca mulatta

## Background & Summary

Using both depth electrode recording^1–6^ and non-invasive brain imaging^7–13^ techniques, a wealth of studies have shown that even in the absence of sensory input from the external environment, certain brain regions tend to share correlated patterns of neuronal activity, known as ‘resting state correlations.’ Such correlations have been observed across multiple sensory areas, such as auditory cortex^14^, visual cortex^1,2,5,9,11,13,15,16^, and somatosensory cortex^6,8,17,18^. They have also been observed in motor cortex^8,18^ and in areas responsible for higher cognitive functions, such as the prefrontal cortex^8,18^ and the parietal cortex^8,10^.

Recent advances in ultra-high-density electrode fabrication and surgical implantation have spurred a surge in large-scale, multichannel recordings in rodents^19,20^, including from multiple brain regions. However, ultra-high-channel-count electrophysiological recording techniques have yet to become widely adopted in non-human primates. Several challenges need to be addressed: electrode implantation requires access to the brain through a craniotomy (or several craniotomies) in the skull, limiting the number of recording sites and their spatial distribution. Existing probes with high channel counts, such as the Neuropixels probes from Imec (Belgium) were developed for mice and are often too fragile for chronic implantation in the primate brain^21^, although more sturdy versions are under development. Presently available probes that are robust enough have relatively modest channel counts. Previous electrophysiological studies in non-human primates therefore usually involved the simultaneous implantation of up to dozens or, maximally, hundreds of electrodes in the brain^22^.

In this study, we developed a novel neuronal recording system and implantable interface, to achieve chronic, high-resolution, large-spatial-scale recordings of neuronal activity in the visual cortex (V1 and V4) of two macaque monkeys^23^. These techniques allowed us to record neuronal activity across 1024 channels simultaneously, with extensive, high-density receptive field (RF) coverage across a large portion of the visual cortex (with overlap between V1 and V4 RFs), spanning the central 6–9 degrees of visual angle across one quadrant of the visual field. Our dataset^24^ covers the full range of spectral components from raw signals sampled at 30 kHz to local field potentials (LFP, at 1–100 Hz) to multiunit spiking activity (MUA, at 500–9000 Hz).

We expect these resting state data to be of interest to neuroscientists in the fields of computational and systems neuroscience. Potential applications include correlation analyses, large-scale modelling^25^, detection of activity waves^26^, teaching material, and more. For example, the strength and anatomical distribution of co-variations in activity could shed light on the anatomical and functional connectivity between or within the areas under examination^27^, including the retinotopic organisation of V1 and V4.

In the visual system, resting state correlations have been used to calculate functional correlations between brain regions in order to identify the borders of visual cortical areas such as V1, V2 and V3^15,28,29^, and for the estimation of the retinotopic layout within individual visual areas^30–32^. Across visual areas, the lateral geniculate nucleus has been observed to exhibit higher levels of correlated activity with primary visual cortex than with higher-order visual areas, whereas activity in V2 and V3 is more closely correlated with V4 and hMT+^11^. Retinotopically corresponding locations across areas V1, V2 and V3 show increased functional connectivity, and a similar pattern has been observed for corresponding brain regions in the two hemispheres.

This dataset could further be used to compare electrophysiologically recorded neuronal activity to that obtained using non-invasive techniques. To give an example, we recently compared population RF estimates obtained with multiple-channel electrophysiology and fMRI-generated BOLD activity^33^. Indeed, MRI^8–10,15^ and invasive electrophysiology^1–5,27,34–37^ provide complementary approaches to examining correlations, including during resting state: fMRI offers a large-scale perspective, revealing the interplay between multiple brain areas and permitting the examination of entire resting state networks, via fluctuations in the MRI signal which have a relatively coarse spatial and temporal resolution. By contrast, electrophysiology yields direct recordings of neuronal activity from a smaller set of brain regions, but at a high spatial and temporal resolution.

The present dataset could also serve as a template for future publications of electrophysiology datasets, providing standardized methods and tools for the description, preparation, and organization of both data and metadata, thereby contributing to the present era of open data sharing and collaboration.

The data are available on the G-Node Infrastructure (GIN, https://gin.g-node.org/), an open-access data-sharing platform. The dataset version described in this publication can be found at https://gin.g-node.org/NIN/V1_V4_1024_electrode_resting_state_data. The dataset follows the FAIR principles^38^, i.e. it is designed to be findable, accessible, interoperable, and reusable.

## Methods

**Fig. 1:**
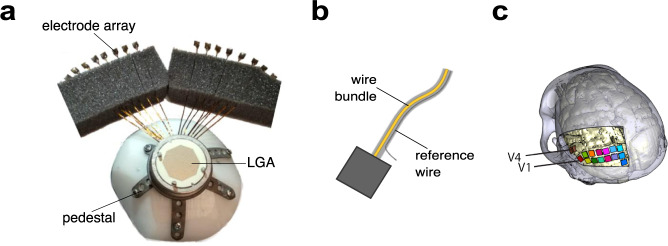
(**a**) Photograph of the implant, consisting of a 1024-channel cranial pedestal connected to 16 Utah arrays. The base of the titanium pedestal was customized to fit precisely on the surface of the skull, as measured with a CT scan. (**b**) The references wires were located on alternating arrays (array numbers 1, 3, 5, 7, 9, 11, 13, and 15) and ran alongside the wire bundle before emerging several millimetres before the point of connection between the wire bundle and the array. (**c**) Location of implantation of every array in the visual cortex in monkey L. Two arrays were implanted in V4, and 14 arrays were implanted in V1, in each monkey.

Our subjects were two male rhesus macaque monkeys (Macaca mulatta, monkeys A and L). Each animal received two cranial implants during two separate surgical procedures. The first of these implants was a customized, in-house-designed, 3D-printed head post for head fixation^39^. The head post was affixed to the setup to stabilize the head throughout the recordings. This ensured that the eye tracker captured the eye data (pupil diameter and position) accurately throughout the recordings (see Chen *et al*., 2017, for a detailed description of these methods). The second was a 1024-channel implant for the visual cortex, consisting of 16 Utah electrode arrays (Blackrock Microsystems) attached via 7-cm-long wire bundles to a customized, in-house-designed, 3D-printed pedestal (referred to in the rest of the manuscript as a ‘1024-channel pedestal,’ Fig. 1)^23^. Each array contained an 8-by-8 grid with 64 iridium oxide electrodes. The length of each electrode shank was 1.5 mm and the spacing between adjacent shanks was 400 μm. The impedance of the electrodes at pre-implantation ranged from 6 to 12 kΩ (as measured by Blackrock Microsystems prior to lead attachment). Each electrode was connected to a contact pad on the Land-Grid-Array (LGA) interface of the pedestal. Reference wires were attached to every other array, and each reference wire served as the reference for two arrays, yielding eight reference wires in total. Each reference wire exited the wire bundle several millimetres before the point where the wire bundle met the array. The other end of the reference wire was connected to one of the contact pads on the LGA of the pedestal (as was each of the electrodes), and referencing was performed by the Cereplex M headstage (i.e. the connections were hardwired such that the electrodes on each pair of arrays used the signal from the reference wire as their reference).

### Surgeries

All experimental surgical procedures complied with the NIH Guide for Care and Use of Laboratory Animals (National Institutes of Health, Bethesda, Maryland), and were approved by the institutional animal care and use committee of the Royal Netherlands Academy of Arts and Sciences (approval number AVD-8010020171046). The subjects were 4 and 5 years old, and weighed 6.5 and 7.2 kg, respectively, at the time of head post implantation; and both were 7 years old, weighing 11.0 and 12.6 kg, respectively, during visual cortex implantation.

A course of antibiotics was started two days prior to each operation. We induced anaesthesia with intramuscularly administered ketamine (concentration of 7 mg/kg) and medetomidine (0.08 mg/kg). We administered 0.1 ml atropine (0.5 mg/ml) if the heart rate dropped below 75 bpm. The animal was placed on a heated mat to allow continuous regulation of body temperature. Eye ointment was applied to maintain hydration of the eyes. Xylocaine ointment was applied to the ear bars of the stereotaxic frame, and the animal’s head was secured in the frame.

For the maintenance of anaesthesia, the animal was intubated and ventilated with 0.8–1% isoflurane (mixed with 60% O_2_ and 40% air) and a catheter was inserted into a vein in the arm. During surgical implantation of the head post, we administered fentanyl at 0.005 mg/kg on indication, Ringer-glucose at 10–15 ml/kg/hour, and antibiotics intravenously. For surgical implantation of the electrode arrays, we additionally administered midazolam at 0.5 mg/kg (concentration 5 mg/ml) once per hour, and we administered dexamethasone at 0.25 mg/kg twice per hour, starting before opening of the skull until skull closure. ECG, heart rate, SpO_2_, CO_2_, temperature, muscle tone, respiration, and the response to pain stimuli were monitored continuously. The head was shaved and cleaned with chlorhexidine solution (Hibicet scrub) and iodine solution (5% iodine in water). For installation of cranial implants, a flap of skin was carefully detached from the skull over the desired implant location, reflected, and wrapped in damp cotton swabs to keep it moist. Methods of implanting the head post and arrays, and post-surgical recovery, are described separately in the following sections.

Head post: We sterilized the titanium head post by autoclaving it prior to surgery. It was placed on the skull and adjusted such that it fitted against the skull. We used 2-mm-diameter Ti cortex screws (DePuy Synthes, Amersfoort, Netherlands) to secure the head post to the bone. The wound margins were sutured together and an extra stitch was made to hold the skin closed around the base of the head post.

**Fig. 2:**
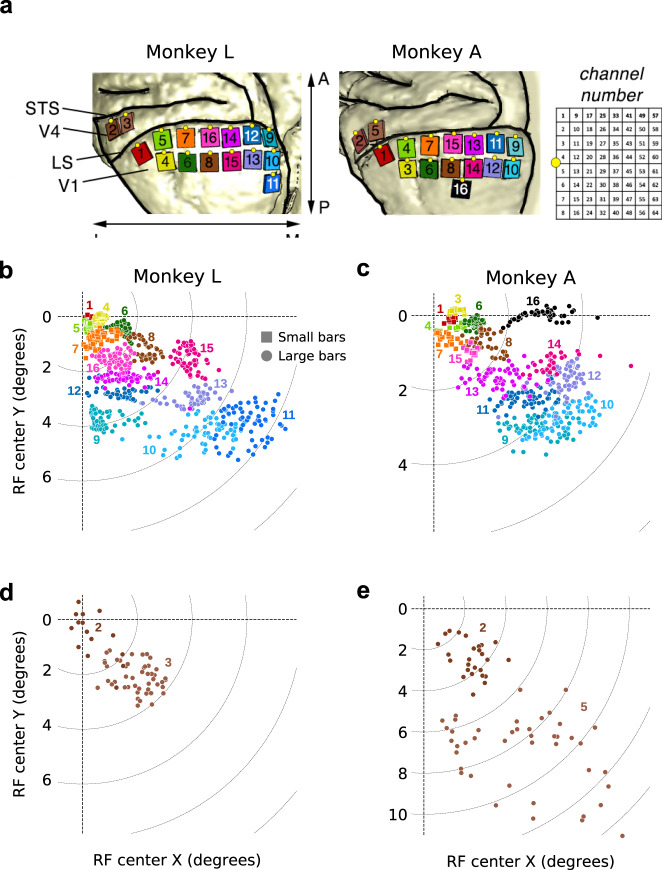
(**a**) Numbering of the 16 arrays that were implanted in the visual cortex. LS: lunate sulcus. STS: superior temporal sulcus. The yellow dot on each array indicates the side on which the wire bundle exits the array. Right: Numbering of channels on each array, as viewed from the top of the array after implantation, rotated 90 degrees CCW relative to the left panel. (**b**–**e**) RF map, showing the coordinates of the V1 (**b**,**c**) and V4 (**d**,**e**) RF centres for channels with an SNR of more than 2 for each condition (*N* = 893 and 679 in monkeys L and A, respectively). Channels are colour-coded by array number, using the same colour code as in a. The receptive fields are located in the lower-right quadrant of the visual field.

1024 channel implant: Before the surgery, we sterilized the implant (Fig. 1a) using gamma radiation. During the surgery the pedestal was placed on the skull and secured with bone screws. We made a craniotomy over the left hemisphere and opened the dura. We implanted 16 arrays of 64 electrodes each in the visual cortex (14 arrays in V1, and 2 in V4; Figs. 1c, 2a). The dura was sutured closed. We filled the space under the bone flap with Tissucol (Baxter) and placed the flap back while the Tissucol was still fluid. We secured the bone flap to the skull with Ti strips. The skin was pulled back around the pedestal and sutured closed.

Ten minutes before the end of the surgery, the ventilator was switched to stand-by mode, allowing spontaneous breathing. Upon conclusion of the procedure, the monkey was released from the stereotaxic frame. The isoflurane was switched off and an antagonist was administered intramuscularly (i.m.) (atipamezole 0.08 mg/kg), allowing the animal to wake up.

Recovery: Subjects were closely monitored following the operation and given several weeks to recover. We administered antibiotics (typically amoxicillin and clavulanic acid) for 10 days (in consultation with a veterinarian) and dexamethasone for five days in decreasing doses (from 0.7 mg/kg i.m. to 0.1 mg/kg i.m.). As analgesia, we initially used Temgesic, at two doses per day (0.003 mg/kg i.m.). After three days we switched to finadyne, once a day, for six days (1–2 mg/kg i.m.). The socially housed animals were housed solitarily during the first 8 to 9 days following surgery, after which social housing was resumed.

At the time of recording, the post-surgical implantation period was 2 and 4 years for the head posts, and 3 months and 1 year for the 1024-channel pedestal, for monkeys L and A, respectively, and the customized implants remained mechanically stable and well anchored to the skull throughout this period.

### Datasets

**Fig. 3:**
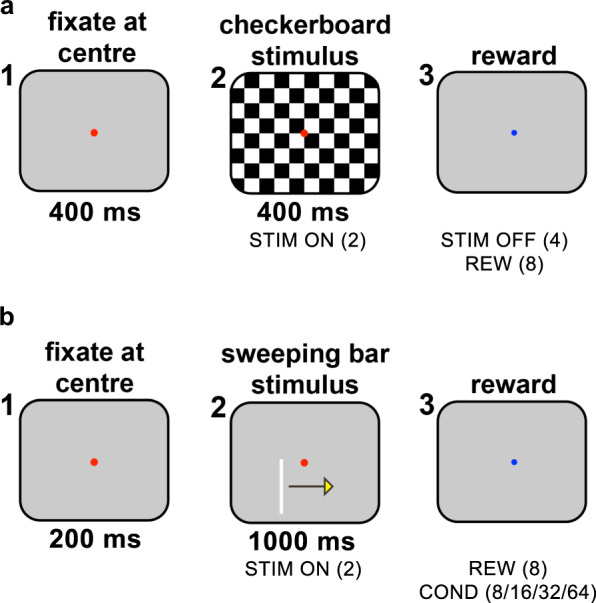
(**a**) Illustration of the task used to measure visually evoked responses and calculate SNRs. 1: The monkey initiates the trial by fixating on a red spot at the centre of the screen. 2: After 400 ms, a checkerboard stimulus is presented. The monkey is required to maintain fixation throughout stimulus presentation, which lasts for 400 ms. 3: The monkey receives a reward upon stimulus offset. Event codes (as recorded in the.nev files) are shown between brackets; e.g. stimulus onset is encoded by the value ‘2.’ (**b**) Illustration of the RF mapping task. 1: The monkey initiates the trial by fixating on a red spot at the centre of the screen. 2: After 200 ms, a bar stimulus is presented, which moves in one of the four cardinal directions (yellow arrow). The monkey is required to maintain fixation throughout stimulus presentation, which lasts for 1000 ms. 3: The monkey receives a reward upon stimulus offset.

In this study, we present 1) resting state data from the two monkeys, collected across three recording sessions per animal. In addition to this main dataset, we collected two supplementary datasets to allow further interpretation of the resting state data: 2) a dataset acquired during a visual fixation task, collected across three recording sessions per animal (on the same days as the acquisition of the resting state dataset), for quantification of the size and signal-to-noise ratio of the neuronal responses elicited by a visually presented checkerboard stimulus (Fig. 3a); and 3) a dataset acquired during a fixation task, collected across two recording sessions per animal, in which we presented moving light bars to map the receptive fields (RFs) of the neurons (Fig. 3b). Table 1 provides a list of all the recording sessions for the three datasets.

**Table 1:** Overview of datasets and sessions.

Monkey	Task type		Recording day (dd/mm/yyyy)	Duration	Good channels (SNR > = 2)
A	Resting state		14/08/2019	32 min 34 s	—
			15/08/2019	38 min 17 s	—
			16/08/2019	42 min 0 s	—
	SNR		04/10/2018(*)	2 min 37 s	899
			14/08/2019	4 min 54 s	359
			15/08/2019	3 min 54 s	416
			16/08/2019	4 min 12 s	379
	RF	Large bars	28/08/2018	18 min 35 s	769(**)
		Small bars	29/08/2018	10 min 42 s	931(**)
L	Resting state		25/07/2017	22 min 42 s	—
			9/08/2017	22 min 0 s	—
			10/08/2017	21 min 37 s	—
	SNR		25/07/2017	1 min 36 s	981
			9/08/2017	1 min 35 s	977
			10/08/2017	1 min 58 s	992
	RF	Large bars	26/06/2017	33 min 43 s	957(**)
		Small bars	28/06/2017	31 min 47 s	821(**)

Channel quality is based on the SNR. For each resting state session, an SNR session was collected on the same day to provide a measure of signal quality. *Extra SNR session, which was collected at an earlier period in time than the other SNR sessions, and which does not have a matching resting state session. **Channel quality in the RF datasets is considered to be good if the channel showed an SNR >  = 2 for any sweeping bar direction.

#### Resting state

For the resting state recordings, the monkey was seated, head-fixed, in a room next to the operator room, with the lights turned off. The room was silent during the recordings (although it was not acoustically isolated). Note that although the lights were off, the setup was not completely dark, due to the presence of small LED lights on our recording equipment, and a small amount of light coming under the door from the adjacent room. The monkey did not carry out a task and was allowed to stay awake or fall asleep at any point in time during the recording, and was free to shift its gaze and centre of attention. We recorded the pupil diameter and the eye camera also allowed us to determine whether the eyes were open or closed.

Our aim was to provide a resting state dataset in which the signals were likely to be derived from the same or similar groups of neurons, allowing for pooling of data across the recording sessions. Therefore, the three resting state sessions were recorded within a short time span (across consecutive working days where possible).

#### Visually evoked activity

For each resting state dataset we also collected a dataset with visually evoked activity on the same day in order to provide an assessment of the quality of the neuronal signal on each channel that day. This dataset consisted of at least 30 trials in which the monkey viewed a grey screen (with a luminance of 14.8 cd/m^2^) before a full-screen checkerboard stimulus was presented for 400 ms while the monkey maintained fixation on a dot located at the centre of the screen (Fig. 3a). The levels of visually evoked activity (relative to baseline activity) provided a measure of the quality of the neuronal signal obtained on each channel. We determined the ‘signal-to-noise-ratio’ (SNR) as the amplitude of the visually driven response divided by the standard deviation of activity in a time window before stimulus onset (see below for details). If desired, the SNR may be used during subsequent analyses to select only the channels from the corresponding resting state dataset that clearly have stimulus-evoked responses and to discard those that show poor or no signal. The size of the checkerboard squares was 1 degree of visual angle (dva), and the luminance values of the black and white squares were 0 and 92.1 cd/m^2^, respectively.

In monkey A, the resting state data and matching visually evoked data were collected 1 year after surgical implantation, following the completion of other (unrelated) experiments. By this time, the number of channels with high SNR had decreased, compared to the number observed soon after surgery. To allow future users of the data to carry out analyses of visually evoked responses across close to 1024 channels (independently of the resting state data), we provide an ‘extra’ dataset of visually evoked activity from monkey A, which was obtained 10 weeks after implantation. This ‘early’ SNR dataset was collected using an identical task design to that of the other SNR datasets, while providing a larger number of channels with high SNR. Note that this additional dataset is stand-alone and is not paired with a resting state session.

#### Receptive field mapping

The subjects viewed moving light bars that appeared at specific locations on the screen, allowing us to identify the RF location of the neurons recorded on each channel.

To characterize the receptive field properties on each channel, we recorded the responses evoked by white sweeping bar stimuli that moved in each of four possible directions (top to bottom; bottom to top; left to right; and right to left)^40^. RF size scales with eccentricity^41^: the farther away an RF is from the fixation, the larger its size. Neurons with small RFs respond best to small stimuli, whereas neurons with larger RFs show a more pronounced response to large stimuli.

Hence, the RF mapping task included two stimulus sets: 1) RFs of low eccentricity were mapped out using a small, thin, slow-moving bar (4 degrees of visual angle [dva] in length, 0.04 dva in thickness, moving at a rate of 4 dva per second) that was positioned close to the fixation spot. 2) RFs of higher eccentricity were mapped out using a long, thicker, faster-moving bar (20 dva in length, 0.19 dva in thickness, 20 dva/s) that was positioned farther from the fixation spot (see Fig. 3b). Stimulus presentation was controlled using custom-written Matlab scripts (Table 2) that were run on the stimulus control computer. The two types of visual stimuli elicited spatially and temporally well-defined neuronal responses, which allowed for the measurement of RFs closer to and farther away from fixation.

**Table 2:** List of Matlab scripts used for experimental control and stimulus presentation, with script names and descriptions.

*Experiment control scripts*		
Task	Script name	Description
Resting state	sync_pulse_resting_state.m	Sending of sync pulses to eight NSPs for post-hoc alignment of raw data.
SNR	runstim_CheckSNR.m	Presentation of full-screen checkerboard stimuli to elicit visually evoked responses.
RF	runstim_RF_barsweep_stimcondition1.m	Presentation of small sweeping bar stimuli to carry out RF mapping on channels where RFs were close to fixation.
	runstim_RF_barsweep_stimcondition2.m	Presentation of large sweeping bar stimuli to carry out RF mapping on channels where RFs were further from fixation.

The experimental control scripts are highly specific to the hardware and are not designed to run without the equipment. We provide the scripts for completeness.

### Data collection

**Fig. 4:**
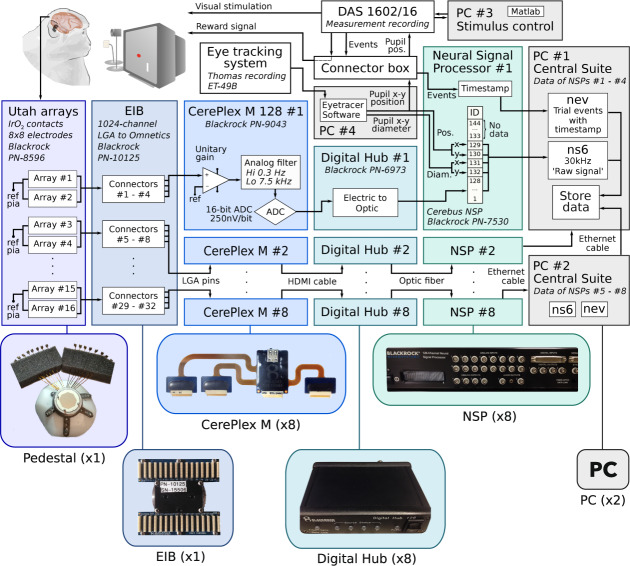
Overview of devices and the total number of units (shown in parentheses) used to obtain, process and store data. Boxes represent individual devices/systems and arrows show the direction of signal transmission between the devices. Note that pairs of Utah arrays ultimately connect to a single neural signal processor (NSP), giving rise to a total of 8 parallel connection pathways. The apparatus and connections for the first connection pathway (i.e. for the first pair of Utah arrays) are shown in detail, while those for the other 7 pathways (for arrays 3 to 16) are depicted in condensed form. Pupil size and position are only recorded on NSP #1, and are not sent to the other 7 NSPs.

Electrophysiological signals from V1 and V4 were recorded from 1024 channels distributed across 16 Utah Arrays (each consisting of 8 × 8 electrodes), at a sampling rate of 30 kHz (see Figs. 1c, 2a for their locations in the visual cortex), and further processed by equipment from Blackrock Microsystems (see Fig. 4 for a schematic overview of the setup). The neuronal signals were passively conducted via the LGA interface on the 1024-channel pedestal to an electronic interface board (EIB), i.e. an adapter with 32 36-channel Omnetics connectors, which in turn interfaced with eight 128-channel CerePlex M headstages. Each CerePlex M processed signals from two 64-channel Utah arrays, applying a 0.3–7500 Hz analog filter at unity gain (i.e. no signal amplification was carried out). The CerePlex M performed a 16-bit analog-to-digital conversion (ADC) with a sensitivity of 250 nV/bit. The digitized signal on each CerePlex M was sent to a 128-channel Digital Hub, i.e. each Digital Hub processed data coming from one CerePlex M, which in turn originated from two electrode arrays. The Digital Hub converted the digital signal into an optic-digital format, which was then sent via an optic-fibre cable to a 128-channel Neural Signal Processor (NSP) for further processing and storage. Each Digital Hub delivered the signal to a single NSP. There were eight NSPs and each NSP processed the data derived from two electrode arrays.

Control of the NSPs was carried out on two PCs (PC #1 and PC #2, running Windows 7 Professional) using the Blackrock Central Software Suite (version 6.5.4), with one instance of the software being run for each NSP, i.e. a total of eight instances of the software ran simultaneously during data acquisition. Each PC was connected to four NSPs, and four instances of the software were run on each PC. Each NSP stored the raw neuronal signals from 128 channels in a single raw data file (corresponding to channels 1 to 128), giving rise to a total of eight raw data files across the eight NSPs. The data recorded from the eight NSPs were temporally aligned as described in the section, ‘Temporal alignment of raw data.’

Due to the high volume of data being processed and stored by each NSP, the onset of recording was controlled manually with a temporal offset of several seconds between NSPs. Before starting the recording on any given NSP, the operator checked to ensure that on-going recordings were running smoothly on the other NSPs, thereby avoiding buffer overflow and dropped packet issues due to system overload at the start of recording. Automatic updates were disabled to prevent unwanted disruptions during recording.

#### Eye tracking

During each recording, an infrared eye tracker (TREC ET-49B, version 1.2.8, Thomas Recording GmbH) was used to sample the eye position and pupil diameter for both the X- and Y-axes with a frame rate of 230 Hz, and the data were stored at a sampling rate of 30 kHz.

The eye tracking hardware was controlled by a dedicated PC (PC #4) using Eyetracer software (Thomas Recording), which forwarded the analog signals regarding the eye position and pupil diameter directly to NSP #1. X and Y eye positions were recorded on channels 129 and 130 of NSP #1, respectively, while X and Y pupil diameter were recorded on channels 131 and 132, respectively.

On all NSPs, the data collected on channels 1 to 128 comprised the neuronal signals. On NSP #1, channels 129 to 132 additionally contained the eye signals. Furthermore, analog synchronization signals were recorded on the NSP channel 144; these can be ignored as the synchronization pulses were also registered as digital events in the.nev files. All other NSP channels (133–143) did not record any data. We stored the raw data from the relevant channels (containing neural and eye signals) as specified in the configuration files that were loaded into the Blackrock Central Software Suite for each NSP.

#### Stimulus and reward timing, stimulus identity and experiment control

A stimulus control computer (PC #3) was used for the execution of task-related events and stimuli with high temporal precision during each of the task paradigms. The task-related event codes consisted of numbers that were sent out from PC #3, via a Data Acquisition and Control System (DAS) Multifunction Analog and Digital I/O board (DAS1602/16, Measurement Computing) through a splitter cable, to the digital input ports (16-bit DB37) on each of the NSPs. The corresponding channels on the digital input port of each NSP sampled the incoming signal at 30 kHz and were configured to detect when an incoming bit was set to a ‘high’ value on one of the pins. In our experiments, only the first 8 digital input pins (1 to 8) on the digital input ports of the NSPs were used, whereas the other 8 digital input pins (9 to 16) were disregarded. To encode a bit change initiated by the stimulus control computer, a 500-ms voltage pulse was sent on the desired pin. On each NSP, the event codes were recorded in the events file (.nev) as a sequence of numbers that ranged in value from 1 to 8. Note that since the DAS board used zero-based indexing, when instructions were sent from the Matlab script to the DAS board, the sequence of pin numbers specified in the Matlab script ranged from 0 to 7, instead of 1 to 8. Table 3 provides a list of the bit identities and their interpretations.

**Table 3:** Relation between bit identity that is sent by the stimulus control computer, the event that is encoded in the events file (.nev) by the NSPs, and the trial-related event that occurred at the moment that the bit was set to ‘high’.

Bit	NEV encoding	Interpretation			
		Resting state	SNR	RF	
				Cond	Description
0	1	Sync pulse	—	—	—
1	2		Stimulus onset	—	Stimulus onset
2	4		Stimulus offset	—	Reward delivery
3	8		Reward delivery	1	Rightward sweeping bar
4	16		—	2	Upward sweeping bar
5	32			3	Leftward sweeping bar
6	64			4	Downward sweeping bar
7	128			—	—

Note that only a single bit is activated at each point in time. The resulting decimal code is 2^*N*, where *N* is the identity of the active bit/pin.

As the precise times at which recording was initiated or terminated varied across the eight NSPs, the duration of the raw data traces also varied slightly between NSPs. Hence, the common digital signal that was sent to all eight NSPs simultaneously via their digital input ports was used to precisely align the raw data traces between NSPs during data processing (described in the section, ‘Temporal alignment of raw data’).

During the resting state sessions, the digital signal consisted of a randomly generated sequence of numbers (ranging in value from 1 to 8), which were sent at 1-second intervals using a custom Matlab script that was run on the stimulus control computer. A list of experimental-control scripts is provided in Table 2. During the SNR and RF mapping tasks, a series of trial-related event codes were sent. Event codes were sent upon stimulus onset and offset and during reward delivery via the same system as that used to send sync pulses during the resting state. Additionally, during the RF task, the stimulus condition used on that particular trial (the direction of bar movement) was sent as an event code. PC #3 also received a copy of the X and Y eye position to check the gaze fixation and determine the success or failure of a trial. Instructions for fluid delivery were then sent to the reward system (Crist Instruments) (Table 3). A summary of the digital codes that were used for the three datasets is provided in Table 2. During post-hoc analysis of the raw data, trial-related events could be identified with high temporal precision (with 30-kHz resolution) and were used for precise temporal alignment of data across NSPs.

### Data pre-processing

**Fig. 5:**
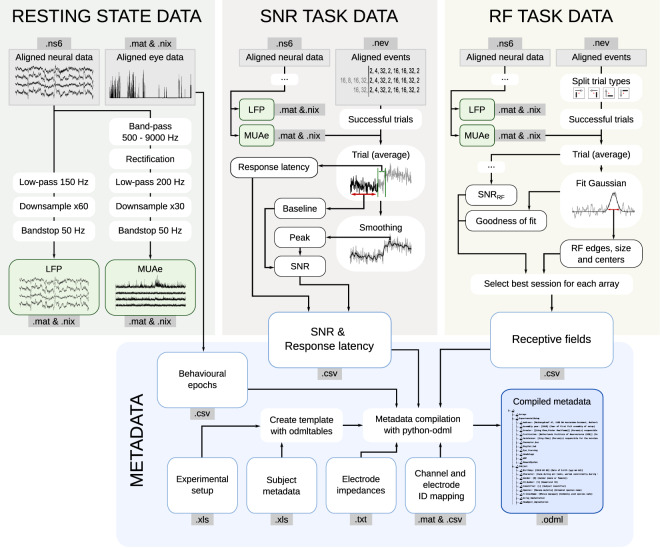
Data pre-processing diagram. Top: Processing steps for the three datasets, leading to the output data and metadata files. Snippets of the full data are depicted for illustrative purposes. Data alignment precedes the processing steps shown here. Bottom: Integration of metadata into a hierarchical odML file. Metadata were both externally collected (recording apparatus, subject-specific metadata, etc.) and calculated from the recordings (eye signal epochs, RF, SNR). All metadata are integrated into a single odML file per session.

The datasets are comprised of temporally aligned raw data, as well as data that have been pre-processed to facilitate their usage. The pre-processing steps included the extraction of local field potential (LFP) signals and envelope multiunit activity (MUAe, which represents the aggregation of spiking activity across multiple units recorded via one electrode – details on how we computed MUAe are provided below)^40^ from the raw recording traces, and a systematic registration of metadata. These steps were executed after the recording session and implemented into a Python workflow using the Snakemake workflow management system^42^. In addition to this fully integrated workflow, standalone Matlab pre-processing scripts are also included. The metadata integration is only provided based on Python. A full list of data-processing scripts is given in Online-only Table 1. See a schematic description of the data pre-processing workflow in Fig. 5.

#### Temporal alignment of raw data

The onset and offset of recording were not synchronous across NSPs. Hence, the raw neuronal data were temporally aligned across the files that were generated by the eight NSPs. Excess data at the beginning and end of each file that were not common to all eight NSPs were removed, yielding files of the same duration. Any channels that did not contain neuronal data, i.e. channels 133 or higher on NSP 1 and channel 129 or higher on NSPs 2 to 8, were also removed. The temporally aligned data were saved in the .nev and .ns6 formats. The unaligned raw data files are not provided in the data repository due to their large volume, but are available upon reasonable request. The lightweight events files (.nev) are provided in both their aligned and non-aligned form.

#### Eye signal processing

**Fig. 6:**
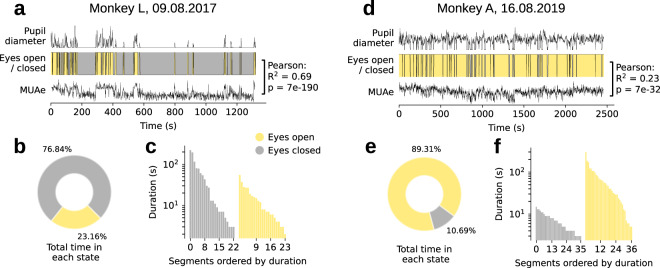
Overview of pupil diameter during an example resting state session, for each monkey. (**a**,**d**) Traces showing the pupil diameter, the state of the eye (‘open’ or ‘closed’) and the mean MUAe from the highest-SNR electrode array. The Pearson correlation between the eye state and MUAe is also shown. (**b**,**e**) Percentage of time spent with eyes open and closed. (**c**,**f**) Bar plot of the duration of time segments that were spent in each state, ordered by duration (segments shorter than 100 ms are likely to be eye blinks and are not shown). Colour coding is identical for all panels.

In many human studies on resting state activity, subjects are given blindfolds and asked to keep their eyes closed. For the resting state sessions in our subjects, we recorded the pupil size and included it in the dataset, instead of using blindfolds. The eye position and pupil diameter (channels 129 to 132 on NSP #1) were temporally aligned, labelled, and saved in .mat and .nix format (Fig. 5). The baseline value of the recordings containing the pupil diameter was not at 0 mV. Hence, this signal was corrected by subtracting its minimum value within the given session. Additionally, we identified whether the eyes were open or closed, i.e. eye closure. We down-sampled the signals to 1 Hz, to reduce noise and exclude short blinks, and combined the X and Y pupil diameter readings using the Euclidean norm. A low threshold was set and if the combined diameter signal fell below this threshold, we considered the eyes to be closed, otherwise they were considered to be open (Fig. 6). During the recordings, the subjects occasionally exhibited signs of sleepiness and their eyelids drooped for a while, before they closed their eyes completely. Their eyes would sometimes stay closed for minutes at a time. These epochs can be found in the eye data, as extended periods in which the pupil diameter is below threshold. Users may for instance select the time periods during which the monkeys’ eyes were closed for a given duration for further analyses of the resting state. We provide both the full (30-kHz) and down-sampled (1-Hz) eye signals in the data repository.

#### Generation of MUAe and LFP signals from raw data

Following the temporal alignment of raw data across the NSPs, two commonly used types of neuronal signals were extracted from the data (Fig. 5): envelope multiunit activity (MUAe) and local field potentials (LFP).

To generate the MUAe, the raw data were filtered between 0.5 and 9 kHz. A full-wave rectification was performed on the filtered signal, followed by a low-pass filter of 200 Hz. Filtering was carried out using a Butterworth filter, of order 4. The data were down-sampled by a factor of 30, yielding for each original signal an MUAe signal with a sampling rate of 1 kHz.

To generate LFP signals, the raw data were low-pass filtered at 150 Hz (Butterworth filer, order 4) down-sampled to 500 Hz. The newly generated MUAe and LFP signals were saved in the .mat and .nix file format, where each file contains the data from one Utah array with 64 channels.

### Signal-to-noise ratio (SNR)

To quantify the signal quality of the recorded neuronal activity, the signal-to-noise ratio (SNR) for each channel was calculated based on the amount of visually evoked activity that was elicited upon presentation of a full-screen checkerboard stimulus, relative to baseline activity, across a minimum of 30 trials. We calculated the mean and standard deviation (SD) of the MUAe during the 300-ms time window prior to stimulus onset (Mean_spontaneous_ and SD_spontaneous_ of the baseline activity) for each trial. Next, trial-averaged MUAe data were smoothed with a moving average of 20 bins (i.e. at a sampling rate of 1 kHz, each bin comprised 20 ms), and we identified the peak level of activity elicited during stimulus presentation (Peak_stimulus_evoked_). The SNR was then calculated following Eq. 1:

1 $$SNR=\frac{Pea{k}_{stimulus\_evoked}-Mea{n}_{spontaneous}}{S{D}_{spontaneous}},$$

A high SNR is indicative of a functional electrode that yields good-quality MUAe. Since the electrodes were located in the visual cortex, they were expected to show responses to visually presented stimuli. A low SNR value may be indicative of one of two situations: 1) For channels where the receptive fields overlap with or are located close to the fixation spot (close to the sulcus between V1 and V4), the presence of the fixation spot in the receptive field may elicit high levels of activity throughout the trial, including during the ‘baseline activity’ period that precedes stimulus onset. This would result in elevated levels of baseline activity and thereby decrease the SNR. 2) The quality of the signal recorded on that particular electrode may be poor due to factors such as electrode failure, connection failure, poor contact between the electrode and the neuronal tissue and/or excessive tissue gliosis around the electrode.

**Fig. 7:**
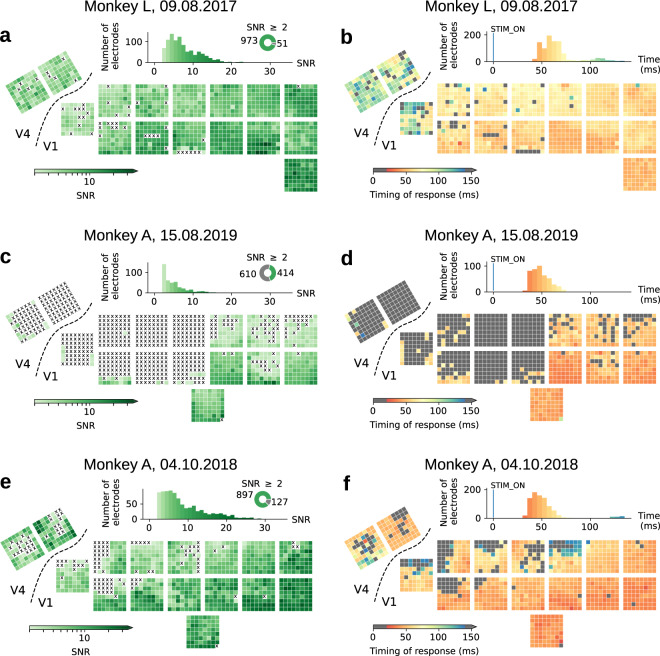
(**a**,**c**,**e**) Channel signal-to-noise ratio (SNR) from an example session for each monkey, shown on a stylized schematic of the arrays on the cortex. Channels with SNR values < 2 are marked with an ‘X’. Top: histogram showing the distribution of SNR values and pie plot showing the proportion of electrodes with SNR > 2. (**b**,**d**,**f**) Stimulus-evoked response timing of two sample SNR sessions, measured as the time at which the trial-averaged MUAe signal exceeds 2 SDs of the baseline (300 ms prior to stimulus onset) for at least 5 consecutive time steps (5 ms in the 1-kHz sampling of MUAe signals). Channels that exhibited a response too early (<20 ms) or too late (>150 ms), or had no response at all, are shown in gray (these channels often had SNR <2). Panels (**a**,**b**) show data from session L_SNR_090817 in monkey L; (**c**,**d**) from session A_SNR_150817 in monkey A; and (**e**,**f**) from session A_SNR_041018 in monkey A. Note that the resting state data in monkey A were recorded several months after surgical implantation of the electrodes, by which time the data quality had decreased (**c**,**d**). Hence, we provide an additional, stand-alone checkerboard stimulation dataset, session A_SNR_041018 (**e**,**f**) which was obtained at an earlier date (when the SNR was high on the majority of channels) and does not have a matching resting state session.

To select good channels for further analysis of MUAe, we recommend setting a threshold value for the SNR (e.g. 2 or higher) to include only the channels with an SNR value that is above the threshold in subsequent analyses. See Table 1 for the number of high-quality electrodes per session and Fig. 7a,c,e for the SNR values from an example session. The SNR values can be found in the metadata files for the corresponding session (Online-only Table 2).

### Neuronal response latency

In addition to the SNR we estimate the neuronal response latency from the checkerboard stimulus task (i.e. the SNR task). We define the response latency as the time elapsed between stimulus onset and the first time that the trial-averaged MUAe signal is more than 2 times the SD_spontaneous_ in 5 consecutive bins. The spontaneous activity period is defined as 300 ms prior to stimulus presentation, as in the SNR calculation. We require the activity to be above the threshold in several consecutive bins to ensure robustness against rapid noise fluctuations. Figure 7b,d,f shows the response latency for a sample session in each monkey. Our measurements are in agreement with previous reports of latency in the visual system^1^. The SNR and latency are calculated together and can be found in the same metadata files (Online-only Table 1).

### Receptive field (RF) mapping

We estimated the RF of each electrode using sweeping bar stimuli. The average MUAe was calculated across trials with a given direction of bar motion. A Gaussian was fitted to this trace, and the onset and offset of the visually evoked response were calculated as the times on each trial that corresponded to the midpoint of the Gaussian minus and plus 1.65 times the standard deviation of the Gaussian, respectively. The vertical and horizontal boundaries of the RF on each channel were then calculated as the mean of two values: 1) the spatial location corresponding to the onset time of the response elicited by a bar moving in a particular direction, and 2) the spatial location corresponding to the offset time of the response elicited when the bar moved in the opposite direction^40^. The x- and y-coordinates of the RF centre were taken as the midpoints between the horizontal and vertical boundaries of the RF, respectively, and the RF size was calculated according to the equation:

2 $$D=\sqrt{{\left(r-l\right)}^{2}+{\left(t-b\right)}^{2},}$$

where *D* is the diameter of the RF, *r* and *l* are the x-coordinates of the right and left boundaries and *t* and *b* are the y-coordinates of the top and bottom boundaries.

The arrays with RFs located closest to the fixation spot (arrays 1 and 4 in monkey L, and 1, 3, 4, 6, 7, 8 and 15 in monkey A) were mapped using a small, thin, slowly moving bar and the other arrays were mapped using a large, thick, fast-moving bar. Note that during stimulus presentation, data were recorded from all the arrays, including from arrays that were not being mapped by the stimulus. Hence, the datasets obtained using the thick and thin bar stimuli were combined into a unified RF map and the remaining data was discarded. The combined RFs for each monkey can be found in the metadata repository (Online-only Table 2).

The RF maps depict the extent of spatial coverage across the visual field. We observed a clear retinotopic organization that matched the locations at which the arrays were implanted on the cortical surface (Fig. 2).

## Data Records

### Identification of array and channel number

As described in the Methods, of the 16 Utah arrays, 14 were implanted in V1 and 2 in V4. Figure 2a shows the location of implantation in the visual cortex for each of the 16 arrays. Each Utah array consisted of 64 electrodes, and each NSP recorded the signals obtained from two arrays, i.e. 128 channels (see the section, ‘Data collection’). Each electrode was assigned a unique global identifier from 1 to 1024. To link the global identity of individual channels with the numbering within an array (out of 64) and the numbering within an NSP (out of 128 channels) we generated look-up tables (LUTs) for each monkey. Each row in the table represents a single electrode. For each electrode the global (out of 1024), within-NSP (out of 128) and within-array (out of 64) channel indices are indicated. Additionally, the NSP number (out of 8), array number (out of 16) and cortical area (V1 or V4) are specified. These tables allow the unique identification of the electrodes across indexing systems.

### Description of file formats

All data can be found at this GIN repository (10.12751/g-node.i20kyh)^24^. The raw, aligned data are provided in the proprietary Blackrock format, .ns6.

The pre-processed signals were stored as .mat and .nix (https://g-node.github.io/nix/) files; .nix files can be loaded using the Python Neo framework^43^ (https://neuralensemble.org/neo/).

Basic metadata from the recording system are saved in the proprietary Blackrock formats,.nev and.ccf. Note that both .nix and .mat data files (listed in Table 4) contain basic metadata; however, for the complete metadata, the metadata files (listed in Online-only Table 2) should be used. All additional metadata files are provided in various machine- and human-readable formats, such as .txt, .xls, .csv and .mat. Metadata were diverse and originated from different sources, such as the experimental equipment, subject specifications, electrode identifiers, signal quality (SNR), receptive fields (RFs), etc. All metadata were organized into a single unified hierarchical structure, using the open metadata markup language (odML)^44^ (https://g-node.github.io/python-odml/), a human- and machine-readable file format for reproducible metadata management in electrophysiology. The raw metadata were processed with odMLtables^45^ (https://odmltables.readthedocs.io) and custom Python scripts. The generated metadata files are listed in Online-only Table 2, all of which are integrated into a single odML file per session.

**Table 4:** Available neuronal and eye signal data files.

*Raw and aligned neuronal data*			
Format	File naming convention	Number of files	Description
.ns6	NSP*X*_aligned.ns6	8 per session	Temporally aligned raw neuronal data files. The length of the data segments is the same across NSPs.
.nev	NSP*X*_aligned.nev	8 per session	Temporally aligned event data files. The duration is the same as in the NS6 files.
	NSP*X*.nev	8 per session	Raw event data files. The duration is the same as in the NS6 files.
***Eye signal data***			
.mat	aligned_eye_data.mat	1 per resting state session	Eye position in horizontal (X) and vertical (Y) coordinates, and pupil diameter in horizontal (X) and vertical (Y) coordinates. Recorded with a sampling rate of 30 kHz, aligned with the neuronal data.
.nix	aligned_eye_data.nix	1 per resting state session	Eye position in horizontal (X) and vertical (Y) coordinates, and pupil diameter in horizontal (X) and vertical (Y) coordinates. Recorded with a sampling rate of 30 kHz, aligned with the neuronal data. The file includes all relevant metadata in the form of annotation dictionaries.
***Processed neuronal data***			
.mat	NSP*X*_array*Y*_MUAe.mat	16 per session	Temporally aligned MUAe neuronal data files, with a sampling rate of 1 kHz.
.nix	NSP*X*_array*Y*_MUAe.nix	16 per session	Temporally aligned MUAe neuronal data files, with a sampling rate of 1 kHz. The file includes all relevant metadata in the form of annotation dictionaries and event epochs.
.mat	NSP*X*_array*Y*_LFP.mat	16 per session	Temporally aligned LFP neuronal data files, with a sampling rate of 500 Hz.
.nix	NSP*X*_array*Y*_LFP.nix	16 per session	Temporally aligned LFP neuronal data files, with a sampling rate of 500 Hz. The file includes all relevant metadata in the form of annotation dictionaries and event epochs.

In the naming conventions, ‘*X*’ represents the NSP number (1 to 8) and ‘*Y*’ represents the array number (1 to 16).

## Technical Validation

### Impedance measurements

Post-implantation, electrode impedance was measured at 1 kHz using the Impedance Tester function in the Blackrock Central Software Suite. These measurements were carried out in the same month that the resting state data were collected, yielding one text file (.txt) per NSP.

These values were subsequently combined across the eight raw data files (one per NSP), yielding a single. csv file that contains impedance data across all 1024 channels. The impedances were also included in the hierarchically organized odML metadata files.

### Eye closure validation

During the resting state sessions, eye pupil diameter and position were tracked using an infrared camera. The monkeys were head fixed throughout the recordings, and the pupil was within sight of the camera at all times, as verified by inspection of the camera feed by an experimenter. To identify the time points with eye closure, we set a threshold for the voltage obtained in the readings for pupil diameter. We further validated this method of threshold setting by comparing levels of cortical activity observed during eye closure and eye opening, and found that activity in the visual cortex was typically higher when the monkeys’ eyes were open than when they were closed.

To carry out this validation, we identified the electrode array that yielded the highest signal-to-noise ratios across all 64 electrodes (monkey L: array 11; monkey A: array 10). We calculated the mean MUAe across electrodes on this array, as a measure of on-going neuronal activity. We observed a high correlation between activity levels and the status of the eye, indicating that eye opening was accompanied by an increase in V1 activity (Fig. 6a,d), and verifying the accuracy of the eye closure analysis.

### Cross-talk removal

An additional analysis was performed on the resting state data in order to assess whether spurious correlations were present. Unexpectedly high correlations could originate from the induction of current via strong external electromagnetic radiation recorded by the electrodes (e.g. power line noise or telecommunications devices) or could be caused by electrical short circuits between two or more electrodes, i.e. cross-talk.

Cross-talk can arise when electrodes physically touch each other due to mechanical bending during or after surgery, or when currents arise between cables due to a breach in electrical insulation of channels at any point along the processing stream up to the conversion of data from analog to digital. We examined the data for spurious correlations, as they could potentially contaminate MUAe signals. Determining the precise source of such spurious correlations is beyond the scope of this publication.

**Fig. 8:**
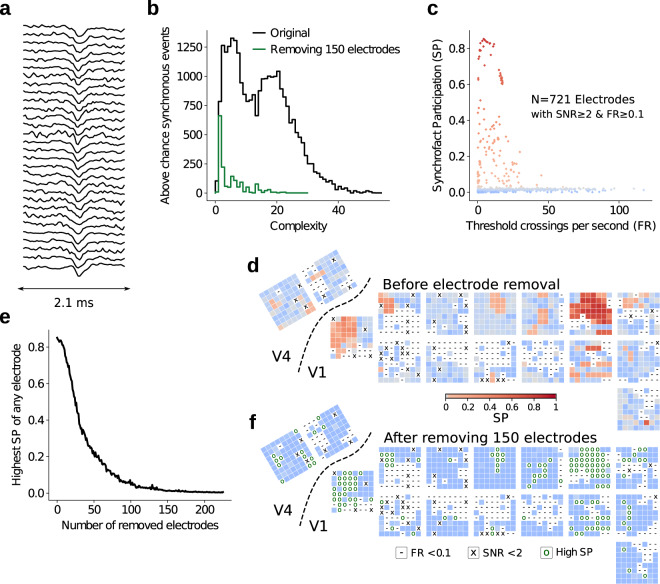
Detection and removal of high-frequency synchronous events. All plots display data from a single resting state session (L_RS_250717), which was the session with the most cross talk. (**a**) Raw signal of a sample synchrofact (complexity = 30). (**b**) Above-chance part of the complexity histogram for the original data (black) and after removing 150 electrodes with the highest synchrofact participation (SP, green). (**c**) Scatterplot of SP of each electrode versus firing rate (FR, calculated as threshold crossings per second). Each point represents a single electrode. As shown by the absence of a positive correlation, the SP was not biased by the FR, due to our method of normalising the SP by the total number of synchronous events. (**d**) Synchrofact participation of each electrode in the original data (prior to removal of 150 electrodes with high SP). Crosses (X) indicate electrodes with SNR < 2 and hyphens (−) indicate electrodes with FR < 0.1; these electrodes were excluded from the cross-talk analysis. (**e**) For the remaining electrodes, those with a high SP were systematically removed, accompanied by a decrease in the largest SP that was observed across the remaining electrodes. (**f**) SP of each electrode after the removal of 150 electrodes with high SP; removed electrodes are indicated by a circle (O). (**c**) (**d**) and (**f**) use the same colour map.

In order to detect cross-talk artifacts in the data, we band-passed the raw signals at 250–9000 Hz. We then removed the first principal component for the channels that shared a common reference wire, roughly corresponding to the mean of the signals. Threshold crossing events were extracted as described by Quiroga *et al*.^46^, with a threshold multiplier parameter of 5. Next, we counted synchronous threshold crossings at sampling resolution and in adjacent sampling bins (1/30 ms). The complexity of a synchronous event was defined as the number of near-simultaneous threshold crossings across electrodes^47^. These synchronous events could also occur in the data by chance. However, in some cases, the analog signals had the same shape across numerous electrodes, indicating that these synchronous events were likely artifacts (see Fig. 8a for an example). These non-random synchronous events are termed ‘synchrofacts’ (short for synchronous artifacts)^47^.

Distinguishing synchrofacts from randomly occurring synchronous events is not trivial, due to the large number (up to hundreds of thousands) of synchronous events, hence we examined their complexity histogram, i.e. the number of detected synchronous events of a given complexity. We used a one-sided Monte Carlo permutation test to check whether the number of synchronous events of a given complexity was above chance level. We generated surrogate data (*N*=1000 surrogates), i.e. permutations, by uniformly dithering (±5 ms) the timing of the threshold crossings. The probability of obtaining a certain number of synchronous events by chance was estimated based on the distribution of events in the surrogate data. We found thousands of synchrofacts across complexity values ranging from 1 to >50 (Fig. 8b), far more than what would be expected by chance.

To distinguish synchrofacts from randomly occurring synchronous events, and to pinpoint the electrodes that were primarily responsible for the non-random events, complexity histograms were then calculated on an electrode-by-electrode basis. To provide a measure of the number of synchrofacts obtained per electrode, i.e. threshold crossings per second (Fig. 8c), we first tallied the number of above-chance threshold-crossing events for a given electrode. Electrodes with higher firing rates inevitably yield a larger number of randomly occurring synchronous events; to correct for this bias, we divided the number of above-chance events on each electrode by the total number of events seen on that electrode. We call this metric the ‘synchrofact participation’ (SP) of the electrode.

3 $$SP=\frac{\sum {N}_{aboveChance}}{{N}_{total}},$$

where *N* denotes the number of synchronous events observed for a given electrode.

The SP takes a value between 0 and 1, indicating the proportion of synchronous events that were above chance for each electrode. Correcting by the total number of events leads to a measure of the synchrofacts per electrode that is not correlated to the firing rate. When mapping the cortical locations of the electrodes with high SP values, we found that they were grouped into several clusters (Fig. 8d). We detected synchronous artifacts in all three resting state sessions from monkey L. Resting state data from monkey A did not show large numbers of synchrofacts (likely due to the low firing rates obtained in those sessions).

The simplest approach to removing cross-talk from the data is to discard the electrodes with high SP from further analysis. We systematically removed electrodes with the highest SP one by one, and recalculated the SP and chance levels after the removal of each electrode. Our significance level was not adjusted for multiple comparisons, as this would lead to a high false negative rate and hinder the removal of electrodes with cross-talk from the dataset. We removed up to 250 electrodes (Fig. 8e), greatly reducing the levels of cross-talk in the data (Fig. 8f). Note that while the electrode removal process eliminates a large portion of the spurious correlations, it does not eliminate artifacts that occur sporadically or at low rates on a given electrode.

We provide a recommended list of electrodes to discard, and the order of their removal. The precise number of electrodes to be discarded can be adjusted as needed by data users, depending on the particular use case. Detailed plots depicting the complexity histograms and electrode SP for all the resting state sessions are available in our data repository.

A reference implementation for synchrofact detection was included in version 0.10.0 of the Electrophysiology Analysis Toolkit^26^ (Elephant, RRID:SCR_003833, https://elephant.readthedocs.io). The full workflow used for systematic electrode removal was implemented with Python and can be found in the data respository.

## Data Availability

All scripts used for the processing of data and our preliminary analyses are available alongside the data at https://gin.g-node.org/NIN/V1_V4_1024_electrode_resting_state_data, in the ‘code’ folder. All experiment control scripts are listed in Table 2 and the data processing scripts are listed in Online-only Table 1. Matlab version R2015b, Python version 3.7 and Snakemake version 5.8.1 were used. The only Matlab dependency was the NPMK toolbox (version 5.0, Blackrock Microsystems), a copy of which is included in the data repository. Direct Python dependencies include neo 0.9.0, nixio 1.5.0 elephant 0.10.0, odml 1.4.5 and odmltables 1.0. A full list of all Python dependencies and the specific versions used can be found in the Python environment specifications, along with the Python scripts (Online-only Table 1).

## Online-only Tables

**Online-only Table 1:** List of Matlab, Snakemake and Python scripts used for data processing and technical validation, with script names and descriptions.

*Matlab data processing scripts*		
Task	Script name	Description
All except impedance data	align_ns6_nev.m	Temporal alignment of raw neuronal data across the NS6 files that are generated by the eight NSPs.
Resting state	generate_processed_resting_state.m	Extraction of MUAe and LFP data from the aligned NS6 files.
SNR	analyse_CheckSNR2.m	Extraction of MUAe data from raw NS6 files, and calculation of signal-to-noise ratio of visually evoked responses.
RF	analyse_RF_barsweep.m	Extraction of MUAe data from raw NS6 files, to identify the peak responses to sweeping bar stimuli.
	analyse_RF_barsweep_coordinates.m	Calculation of RF properties using MUAe data.
	combine_best_RF_sessions.m	Combination of RF data across two sessions, in which different types of bar stimuli were presented.
	plot_all_RFs_resting_state.m	Generates figures showing the RF centres across all the channels.
-	impedance_plotter.m	Compile impedance measurements across all NSPs.
***Snakemake workflow scripts***		
**Script name**		**Description**
Snakefile		Main workflow file, executes all other scripts to generate the processed data and figures.
environment.yaml		Conda environment file. Contains full list of all Snakemake and Python dependencies. Can be used to recreate the Conda environment that was used to run the workflow.
***Python data processing scripts***		
**Task**	**Script name**	**Description**
All	calculate_LFP.py	Calculates the LFP signal from a given array, annotates it with the corresponding metadata and saves it as one .nix file per array.
	calculate_MUA.py	Calculates the MUAe signal from a given array, annotates it with the corresponding metadata and saves it as a .nix file per array.
	utils.py	Several utility functions for the handling of metadata and annotation that are used by the other scripts.
	initialize_odml_from_xls.py	Creates a template .odml metadata file that contains the information of the experimental setup and specific subject.
	enrich_odml_IDs.py	Incorporates subject specific electrode IDs to the template .odml metadata file.
	enrich_odml_epochs.py	Includes subject and trial specific epoch/trial metadata such as the recording length, number of epochs (open or closed eyes in resting state sessions) or number of successful/failed trials (in SNR and RF tasks), as well as their timing and duration.
SNR & RF	generate_trial_csv.py	Generates a .csv file containing the trial start times, durations, conditions and whether they were successful. This .csv file is used by the enrichment scripts to incorporate this metadata into the .odml format.
	merge_csv.py	Utility function that merges a list of .csv files. Used to merge metadata.csv files from RF and SNR values from all different arrays into one.
SNR	calculate_SNR.py	Calculates the signal-to-noise ratio (SNR) from the MUAe.nix data of a checkerboard stimulus session and saves it as a .csv file, along the response amplitude, response onset and baseline specifications.
	arrayplot_SNR.py	Creates the plots in Figure 7a,b for each SNR session.
	arrayplot_response_timing.py	Creates the plots in Figure 7c,d for each SNR session.
	finalize_odml_SNR.py	Aggregates all metadata for a given SNR session and creates the final.odml.
RF	calculate_RF.py	Calculates the receptive field (RF) responses from the MUA.nix files of a corresponding session and generates a .csv file with RF centers, edges, signal-to-noise ratios and the goodness of the Gaussian fit.
	combine_RF.py	Combines the large and small bar receptive field (RF) responses into a single metadata file by selecting the best arrays from each session.
	finalize_odml_RF.py	Aggregates all metadata for a given RF session and creates the final .odml. The combined RF metadata are used for both large and small bar RF sessions.
Resting state	eyesig_conversion_and_epochs.py	Converts the eye signals from .mat to .nix and estimates when the eyes were open or closed from the pupil diameter. The estimated epochs are save as.csv files.
	eye_epochs_plot.py	Creates the plots in Fig. 6 for each resting state session.
	finalize_odml_RS.py	Aggregates the corresponding SNR and RF values to the metadata of a resting state session and creates the final .odml. SNR values from the same day and combined RF values are used.
Cross-talk removal	highpass_ns6.py	Filters the raw data for extraction of threshold crossings. This file is found in the signal processing folder, but only used for cross-talk removal.
	get_thr_crossings_mpi.py	Extracts threshold crossings from filtered raw signal.
	count_synchrofacts.py	Estimates the presence of synchrofacts by creating complexity histograms from the threshold crossings and surrogates.
	systematic_removal_of_electrodes.py	Removal of electrodes with highest above-chance synchronous events and plotting of the synchrofact process.
	utils.py	Utility functions for cross-talk removal.

**Online-only Table 2:** Metadata and their file formats.

*Experimental setup and subject metadata*			
Format	File naming convention	Number of files	Description
.xls	equipment_specifications.xls	1	List of all hardware used in the recording of the neuronal data.
	subject_*SUBJ*.xls	1 per subject	Subject specifications, such as species, age, initial, surgical procedures and training status.
.csv	channel_area_mapping_*SUBJ*.csv	1 per subject	Mapping of channels across array based (1 to 64), NSP based (1 to 128) and global indexing (1 to 1024).
	approximate_array_positions_*SUBJ*.csv	1 per subject	Approximate relative position of arrays with respect to array 1, and their rotation. Used for plotting.
	elec_position_in_array.csv	1	Position of electrodes within an array relative to the centre of the array. Used for plotting.
***Technical validation metadata***			
**Format**	**File naming convention**	**Number of files**	**Description**
.txt	impedance_NSP*X*_*DDMMYY*.txt	8 per impedance session	Electrode impedance values, measured at 1 kHz.
.mat	impedanceAllChannels_*SUBJ*_*DDMMYY*.mat	1 per impedance session	Impedance data files containing the impedance values for each of the channels.
.csv	*SUBJ_RS*_*DDMMYY_*crosstalk_removal_metadata.csv	1 per resting state session	List of electrodes to be removed due to crosstalk.
***Session metadata***			
**Format**	**File naming convention**	**Number of files**	**Description**
.ccf	NSP*X*.ccf	8 per session	Blackrock configuration files for the NSPs.
.csv	epochs_*SUBJ*_*TASK*_*DDMMYY*.csv	1 per session	Metadata on the different epochs during the recording session. For SNR and RF the trial onset, duration and success is registered. In resting state sessions whether the eyes were open or closed is indicated.
***SNR metadata***			
**Format**	**File naming convention**	**Number of files**	**Description**
.csv	NSP*X*_array*Y*_SNR.csv	16 per SNR session	SNR values, along with baseline mean and SD, peak response and neuronal response latency. The global electrode ID (1 to 1024) is provided to identify the electrodes.
	full_SNR.csv	1 per SNR session	A merged version of all NSP*X*_array*Y*_SNR.csv files.
***RF metadata***			
**Format**	**File naming convention**	**Number of files**	**Description**
.csv	NSP*X*_array*Y*_RF.csv	16 per RF session	SNR values, RF edges, and RF centre coordinates.
	fullRF.csv	1 per RF session	A merged version of all NSP*X*_array*Y*_RF.csv files.
**Compiled metadata**			
**Format**	**File naming convention**	**Number of files**	**Description**
.odml	metadata_*SUBJ*_*TASK*_*DDMMYY*.odml	1 per session	A compiled file of all the metadata for a given session.

In the naming conventions, ‘*X*’ represents the NSP number (1 to 8), ‘*Y*’ represents the array number (1 to 16), ‘*SUBJ*’ represents the subject name (L or A), ‘*TASK*’ refers to the task codename (resting state, SNR or RF), and ‘*DDMMYY*’ is the date of the corresponding session.
